# Aujeszky’s Disease and Hepatitis E Viruses Transmission between Domestic Pigs and Wild Boars in Corsica: Evaluating the Importance of Wild/Domestic Interactions and the Efficacy of Management Measures

**DOI:** 10.3389/fvets.2018.00001

**Published:** 2018-01-24

**Authors:** François Charrier, Sophie Rossi, Ferran Jori, Oscar Maestrini, Céline Richomme, François Casabianca, Christian Ducrot, Johan Jouve, Nicole Pavio, Marie-Frédérique Le Potier

**Affiliations:** ^1^Laboratoire de Recherche sur le Développement de l’élevage (LRDE), INRA, Corte, France; ^2^Laboratoire Interdisciplinaire, Sciences, Innovations, Sociétés, INRA, Marne-la-Vallée, France; ^3^Unité sanitaire de la Faune, Office National de la Chasse et de la Faune Sauvage, Gap, France; ^4^CIRAD, UMR ASTRE (Animal, Santé, Territoires, Risque et Environnement), Montpellier, France; ^5^ASTRE, University of Montpellier, CIRAD, INRA, Montpellier, France; ^6^Laboratoire de la rage et de la faune sauvage, ANSES, Nancy, France; ^7^UR346 EpiA, Centre de recherche Auvergne Rhône Alpes, INRA, Saint Genes-Champanelle, France; ^8^UMR Lieux, Identités, Espaces et Activités (LISA), CNRS-Université Pascal Paoli, Corte, France; ^9^UMR 1161 Virology, Animal Health Laboratory, ANSES-INRA-ENVA, Maisons-Alfort, France; ^10^Unité Virologie Immunologie Porcines, Laboratoire de Ploufragran/Plouzané, ANSES, Ploufragan, France

**Keywords:** *Sus scrofa*, wild boar, interface, virus transmission, Aujeszky’s disease, hepatitis E, Corsica

## Abstract

Wildlife species as reservoirs of infectious pathogens represent a serious constraint in the implementation of disease management strategies. In the Mediterranean island of Corsica, the dynamics of hepatitis E virus (HEV) and Aujeszky’s disease virus (ADV) are suspected to be influenced by interactions between wild and domestic pigs. To improve our understanding of these influences, we first compared the seroprevalences of both viruses in domestic pig populations from different locations with contrasted levels of wild–domestic interactions, ADV vaccination, biosafety, and farm husbandry. Second, we performed an analysis at a more restricted geographical scale, to assess the matching of ADV or HEV prevalence between sympatric wild boar and outdoor pig farms most exposed to interactions with wildlife. Logistic models were adjusted to the observed data. A high seroprevalence of HEV (>80%) and ADV (40%) in pigs, with no significant difference according to the region, confirms that both pathogens are enzootic in Corsica. Vaccination against ADV had a strong protective effect, even when performed voluntarily by farmers. Farm biosafety had an additional effect on pigs’ exposure, suggesting that contact between wild boars and pigs were involved in disease transmission. A strong correlation in HEV seroprevalence was observed between pigs and wild boars that were in close contact, and significantly lower seroprevalence was observed in pigs when they had little contact with wild boars due to spatial segregation. These results suggest a regular HEV circulation between sympatric wild boar and domestic pigs. The high HEV seroprevalence observed in domestic pigs (>80%) suggests a spillover of the virus from domestic to wild populations through environmental contamination, but this hypothesis has to be confirmed. Conversely, even though avoiding sows’ release on pasture during estrus showed some protecting effect in the free ranging pig farms regarding ADV, ADV seroprevalence was not dependent on the swine populations (wild or domestic) or on the wild–domestic spatial overlap, suggesting two quasi-separate enzootic cycles. This information will prove useful for designing more efficient disease management strategies in Corsica and similar contexts.

## Introduction—Context: Two Populations, Two Pathogens, and Multiple Stories

Interactions between wild and domestic animals can play a role in the maintenance of pathogens and thereby compromise the efficiency of disease control strategies (1–3). Contacts may rely on direct (e.g., mating or fighting) and/or indirect transmission routes (e.g., sharing the same contaminated habitat) and can be influenced by human activities such as farming or hunting (4, 5). In extensive outdoor farming areas, wild/domestic interactions can be facilitated by some farming practices that expose domestic animals to contacts with wildlife (6). Similarly, hunting practices can influence the spatial distribution of game populations and their interactions with domestic animals. Wild and domestic swine are particularly at risk of inter-population transmission because they belong to the same species and share the same community of potential pathogens (4, 5, 7). In this context, certain farming practices (use of shared pasture areas, reduced surveillance of the herd, etc.) can facilitate interactions and therefore, have a strong influence on the transmission and circulation of pathogens. On the other hand, biosecurity measures can prevent transmission from wild boars to domestic pigs in high-risk areas (8). The reproductive management of domestic sows during the estrus period can influence the occurrence of sexual interactions between wild boars and domestic females or fights between wild and domestic boars (6, 9). The risk of transmitting pathogens is considered the highest in areas with traditional extensive farming and can determine the dynamics of emerging or reemerging pig diseases (10).

In Corsica, a French Mediterranean island, traditional extensive outdoor pig farming systems remain common. Based on the use of local resources (pastures, chestnut, and oak forests), these systems produce high-quality processed meat for the local and national market. However, this kind of farming facilitates contacts with an important wild boar population, sharing pathogens such as the Aujeszky’s disease virus (ADV) (11–14), hepatitis E virus (HEV) (15–18), or bovine tuberculosis (19). In addition, serious threats such as African swine fever, which is endemic in the neighboring island of Sardinia (20), increase the need to understand and manage wild–domestic pig interactions (17, 21).

The objective of this study is to explore the influence of different farming practices (biosafety, use of natural pastures/forests, ADV vaccination, and reproductive management) and individual factors (phenotype, age, sex, etc.) on the prevalence of two pathogens (ADV and HEV) known to circulate within and between domestic and wild pig populations in Corsica. These pathogens have different transmission routes, being able to infect new susceptible hosts through direct (requiring physical contact) or indirect (not requiring close proximity) interactions. ADV, which remains enzootic among domestic pigs and wild boars on the island (22), can be transmitted by close contact including mating (23–25). In this case, the absence of reproductive management of sows can play a role on its transmission between wild and domestic populations. Conversely, HEV is a pathogen widely spread either by direct contact or through a contaminated environment, which is known to be common in free ranging populations of domestic pigs, wild boars and cross bred animals in Corsica and many other European countries (16, 17). We assumed that the dynamics of both pathogens can be influenced by different degrees of interaction at the wild/domestic interface and domestic pig management practices implemented by farmers.

To test these hypotheses, we implemented a two-step approach to investigate different risk factors. Initially, the seroprevalence of ADV and HEV was analyzed in the domestic pig population in a sample of farms across Corsica, to study the effect of the farm biosafety level, vaccination against ADV and the distribution of these two pathogens among different pig production systems and microregions in Corsica. Subsequently, we focused on traditional pig farms exposed to wild boar populations, located within one particular microregion from north-central Corsica (the Boziu-Verde microregion), to better determine the effect of the spatial interface between the two swine populations (through the shared use of natural pastures and forests) and the protective effect of excluding sows in estrus from natural pastures.

## Material and Method: Collecting Data on Seroprevalence and Farming Systems in a Double Scaled Approach

To cover the potential diversity of extensive farming practices existing in Corsica, our study design incorporated an initial large scale, across-island approach to capture this diversity, and then focused on one specific microregion to assess the interaction between domestic pigs and wild boars (see Study Design: A Two-Step Study on Wild Boar/Domestic Pig Interactions in Corsica). We thus combined different sampling procedures (see Serological Data) to build three datasets for statistical modeling (see Dataset Construction and Data Analysis).

### Study Design: A Two-Step Study on Wild Boar/Domestic Pig Interactions in Corsica

#### Domestic and Wild Swine Populations

Corsica is a French Mediterranean island characterized by a sparse human population (32 inhabitants/km^2^) and an economy principally based on tourism. Pig production is based on extensive outdoor systems, partly relying on pasture resources (acorns and chestnuts), using local and common breeds (*Nustrale* pigs, *Large White*, or *Duroc*), and generating products for several types of markets (“Protected Designation of Origin” products with high added value and short supply chains with direct sales). Pig farmers are thus breeders, but also processors and retailers of their own production as described in several other localities in the Mediterranean area (26). Pigs are slaughtered between November and March after the autumnal finishing period. However, although traditional farming systems are prevalent in Corsica, farming practices may differ greatly across the 300 registered farms, especially regarding epidemiological interactions with wild boars (21, 27).

On this mountainous island, extensively covered by grazing lands with typical Mediterranean vegetation (scrub, bushes, small trees, oak trees, etc.), the wild boar density is estimated to be very high, with around 30,000 wild boars being hunted every year (28). Because the wild and domestic populations share the same resources on the same areas, and sometimes at the same moment, this situation is of key interest to study infectious interactions between domestic and wild pigs.

#### Two-Step Study: Large-scale Approach and Microregional Focus

Both virus dynamics may rely on farming practices, such as biosecurity measures (e.g., fences), vaccination against ADV, and characteristics of the production system: age of slaughtering/hunting (farmers choose the age according to the quality of production output, hunters usually shoot older wild boars, etc.), mating management or spaying females (i.e., farmers may avoid encounters between the sow in estrus and male wild boars either by organizing sow mating before releasing them on pastures or by spaying them). These key factors were included in our datasets to be analyzed (see below). To capture the role of farming practices on pathogen dynamics, we combined two different approaches.

##### Step 1: Study Based on Sampling in Slaughterhouses (Dataset 1)

**Figure 1:**
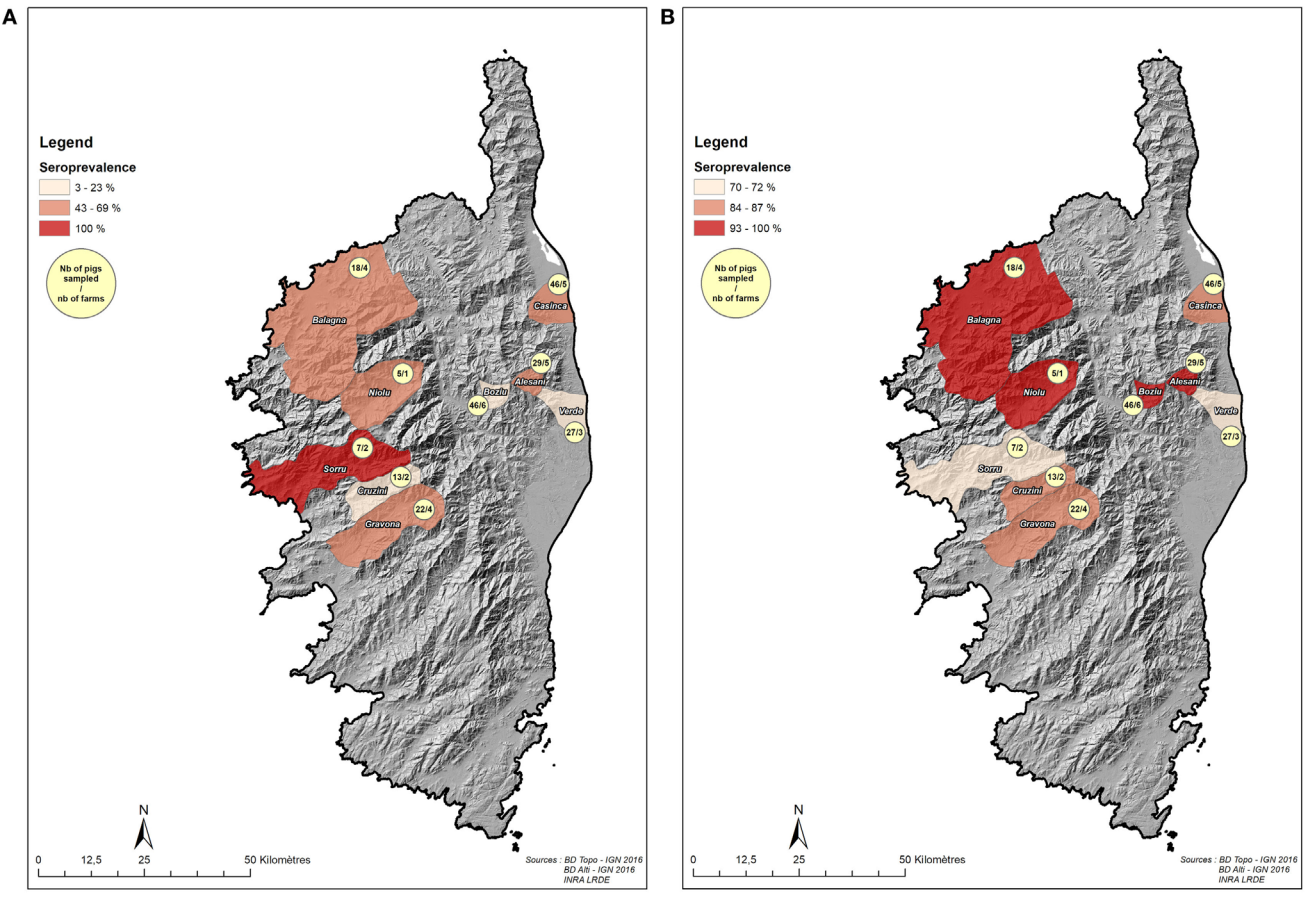
Localization of sampled pigs sampled at the slaughterhouse (dataset 1). **(A)** Number of sampled pigs (slaughterhouse, dataset 1) at the scale of the nine samples microregions and Aujeszky’s disease virus seroprevalence. **(B)** Number of sampled pigs (slaughterhouse, dataset 1) at the scale of the nine samples microregions and hepatitis E virus seroprevalence.

The purpose of this part of the study was to determine the seroprevalence of both pathogens and their geographical distribution and to characterize the potential existing risk factors due to farming practices. To do so, we sampled domestic pigs opportunistically at slaughterhouses to obtain a spatially diversified picture of disease exposure from different areas of Corsica. This sample which was purposive and mainly driven by pragmatic opportunities included 213 pigs originating from 32 farms from several pig production areas across Corsica (Figures 1A,B): investigators collected blood samples during the slaughtering procedure and interviewed farmers when they came to slaughterhouses to bring their pigs or pick up the carcasses. Ten visits to the abattoirs were carried out during the main slaughtering period (December 2014–January 2015) to gage the overall status of each pathogen (HEV and ADV) and to identify the farming practices that play a role in pathogen dynamics.

##### Step 2: Study Based on a Focus on a Particular Microregion (Datasets 2 and 3)

**Figure 2:**
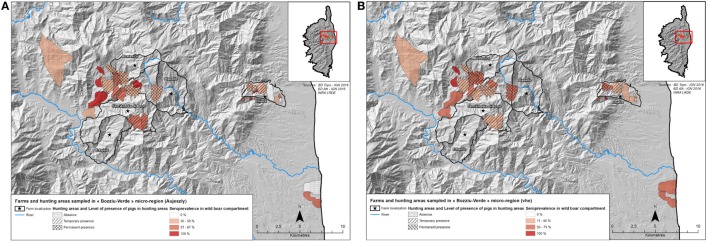
Localization of sampled pigs and wild boars in the “Boziu-Verde” microregion (datasets 2 and 3). **(A)** Localization of domestic pigs sample and hunting areas with Aujeszky’s disease virus seroprevalence. **(B)** Localization of domestic pigs sample and hunting areas with hepatitis E virus seroprevalence.

The purpose was to produce knowledge on pathogen seroprevalence and distribution in domestic pig and wild boar population, targeting traditional outdoor pig farms, supposed to be at risk, from the Boziu-Verde microregion (cf., Figures 2A,B). Six farms were particularly selected for their irregular participation in vaccination campaigns (none of them participated to Aujeszky disease management plan implemented in 2011). Only one farm implemented techniques aiming at reducing risk of contact with wild boars (by female castration and reproduction management), and three of them were known to implement “informal” farming practices (i.e., unofficial farmers and on-farm slaughtering). By focusing on these farms, we hypothesized that the common extensive farming practices (i.e., the regular use of natural pastures) maximized the risk of pathogen transmission between both wild and domestic swine populations. We thus collected data on ADV and HEV seroprevalences and farming practices in pig farms, as well as ADV and HEV seroprevalence in wild boar populations (blood samples collected during hunting sessions, from August to January).

### Serological Data

#### Sample Collection

To study the seroprevalence of these two diseases in the wild and domestic populations, serum samples were collected from wild boars and domestic pigs. For wild boars, blood samples were collected by hunters by means of cardiac puncture on recently shot animals. Blood samples from domestic pigs were collected by veterinarians at the slaughterhouse or by technicians in the herd by blotting filter paper with a drop of blood from the tail of sows.

Blood samples and dried filter papers were sent within 1 day to the INRA research facility in Corte to be centrifuged or dried, respectively. All sera were stored at −20°C, and dried filter papers were kept at 4°C until analysis.

#### Serological Methods

For ADV, all sera and filter papers were tested using an ELISA for the specific detection of antibodies to the ADV gB protein (IdVet-ID Screen^®^ Aujeszky gB competition), according to the manufacturer’s instructions. Because pigs could have been vaccinated with a gE-deleted vaccine, the sera collected from the slaughterhouse were further tested with an ELISA gE (Idexx PRV/ADgI) when they tested gB positive in a vaccinated herd. These two commercial kits have been approved by the ANSES-Ploufragan OIE reference laboratory because they can detect, respectively, the ADV-1 international serum standard at the dilutions of 1:2 for ELISA gB or 1:8 for ELISA gE.

The detection of anti-HEV antibodies in wild boars and domestic pigs was performed using the HEV ELISA 4.0v kit (MP Diagnostics, Illkirch, France) according to the manufacturer’s instructions, except the serum quantity used, 10 µL instead of 20 µL. For detection on filter paper, elution was performed in washing buffer. This sandwich ELISA allows the detection of all antibody classes (IgG, IgM, and IgA) and uses a recombinant antigen that is present in all HEV strains. Samples were positive when the optical density at 450 nm wavelength obtained for the sample was higher than the threshold defined as the mean for negative controls +0.3 for serum and +0.4 for filter paper.

### Dataset Construction and Data Analysis

#### Datasets

Three datasets were considered for the statistical analyses:

(1) *Dataset 1*: A total of 213 domestic pigs from 10 different microregions and 32 farms were sampled in slaughterhouses to assess the distribution of HEV and ADV in different parts of Corsica. Pig sera from vaccinated farms were tested for the presence of antibodies to gB and gE using the two ELISA tests to differentiate between vaccinated (gB+/gE−) and infected (gB+/gE+) animals. Pigs with two consecutive positive results were considered as exposed to ADV (i.e., seropositive), and those testing negative for gB or positive for gB but negative for gE were considered as non-infected (i.e., seronegative). In farms that did not vaccinate their pigs, only gB ELISAs were performed and all pigs with gB-positive results were considered as naturally infected with ADV (i.e., seropositive).
(2) *Dataset 2*: 80 domestic pigs were sampled on 6 traditional farms located in the Boziu-Verde microregion; these farms were not participating in the official vaccination plan and free ranging pigs shared natural pastures/forests with wild boars. For this sample, only gB ELISA was performed since no vaccination could interfere with surveillance.
(3) *Dataset 3*: A total of 297 wild boars were randomly sampled at seven different locations within the Boziu-Verde microregion during five hunting seasons, from 2009 to 2016. 198 sampled wild boars came from the four areas adjacent to farms sampled in dataset 2, and the 99 others came from the three areas that had no or limited contact with pigs (Figures 2A,B). Out of these 297 wild boars, 115 were young (i.e., less than 12 months old). For this sample, only gB ELISA was performed because wild boars are not vaccinated. This dataset is thus composed of 297 wild boars, randomly sampled in the Boziu-Verde area.

#### Dependent and Independent Variables

##### Individuals

For both pigs and wild boars, serological data were categorized as positive and negative results (some rare doubtful results were removed) depending on the detection of ADV and HEV antibodies (dependent variables). The age of domestic pigs was determined by ear tags, whereas the age (young or adult) of wild boars was determined by tooth eruption patterns (29) and body size. Sex and the presence or absence of hybrid phenotype were also recorded. In boars, the presence of colors other than black or dark brown in the coat and the shape and length of the ears were considered as indicators of hybridization with domestic pigs (30). The “hybrid” category thus extended beyond just F1 generation.

##### Farms

For each pig farm, data were collected on its localization (see Figure 1), the level of biosafety of the pig farming area (three levels: free ranging, fenced pastures, and closed piggery), the vaccination treatment (three levels: no vaccination, unofficial vaccination, and official vaccination plan), spaying of females (two levels: yes and no), the reproductive management of female during estrus (two levels: yes and no). Among the six traditional farms sampled in dataset 2, we also recorded the use of natural pastures/forests (two levels: seasonal and permanent) and the type of animal [two levels: reproductive animals (breeder pigs) and pigs intended for meat production (fattening pigs)].

##### Hunting Areas

Hunting areas were classified according to the frequency of domestic pig presence during the year. Three types of hunting areas were distinguished according to hunters’ observations: absence of pigs (no pigs at any time of the year); permanent presence (pigs are spotted on the area all year round); intermittent presence (pigs are spotted only during a specific period, autumn, and when they feed on chestnuts and acorns). Thus, each sampled wild boar was associated with a hunting area (28 areas), which was associated with a variable (3 levels) taking into account the proximity of domestic pigs.

#### Statistical Model

Individual serological results were encoded as 0 (seronegative) or one (seropositive). We used general linear model with logit link to test the risk factors of seropositivity for swine regarding both ADV and HEV. We also tested general mixed models, accounting for the random effects of farm (for pigs) and hunting sector (for wild boars) (data not shown). Since this approach did not provide any improvement in model fit, we finally selected the simplest general linear model approach.

Model selection was based on the Akaike information criterion corrected for over-dispersion and small sample size (QAICc). We performed a preliminary correlation analysis of explicative factors that revealed an important correlation between sow castration and the management sows during estrus in the first data set, so that we finally only tested the female castration treatment as a risk factor in the first step analysis. Starting from a “complete” model including all the potential explanatory variables for the different datasets (detailed in Table 1), we explored simpler models (using the dredge function of the R package MuMin). Considering the QAICc of all the potential sub-models, we used a model-averaging procedure to account for uncertainty in the model selection, because our data corresponded to a small sample size exposed to uncontrolled sampling processes (at the slaughterhouse or of voluntary farms). That means that we could not necessarily retain a “single best model” but rather, a set of potentially “best models” for which the increase in QAICc (delta-QAICc) was less than 2 (31). We finally calculated variable coefficients [i.e., odd ratios and 95% confidence intervals (CIs)] over this set of “best models” according to the process described by Burnham and Anderson (31) (and using the AICmodavg R package). These analyses were performed using R software (32), and the MuMin (33), and AICmodavg packages (34).

**Table 1:** Description of the models and variables tested for each dataset.

(a) Complete model equations				

Disease	Dataset	Number of animals	Complete model equation	
Aujeszky’s disease virus (ADV)	1	151 pigs large scale	Age + castration + microregion + seroHEV + sex + farm type + vaccination	
	2	75 free ranging pigs (Boziu-Verde)	Age + crossbred + pasture use + owner + sow release + seroHEV + sex + repro status + zone	
	3	274 wild boar (Boziu-Verde)	Age + year + hybrid + seroHEV + sex + zone	

Hepatitis E virus (HEV)	1	151 pigs large scale	Age + castration + microregion + seroADV + sex + farm type	
	2	75 free ranging pigs (Boziu-Verde)	Age + crossbred + pasture use + owner + sow release + seroADV + sex + repro status + zone	
	3	274 wild boar (Boziu-Verde)	Age + year + hybrid + seroADV + sex + zone	

**(b) Explicative variables description**				

Subspecies	Individual/pop	Variable	Classes	Datasets
Pig or wild boar	Individual	Age	Young/adult	1–3
Pig or wild boar	Individual	Sex	Male/female	1–3
Pig or wild boar	Individual	Hybrid status	Yes/no	2 and 3
Pig	Individual	Reproductive status	Reproducer/fattening	2
Pig/wild boar	Individual	Serological status for HEV or ADV	0/1	1–3
Pig	Farm	Farm type	Closed/fenced pasture/traditional free ranging	1
Pig	Farm	Vaccination	Official plan/self vaccination/no vaccination	1
Pig	Farm	Castration or sows in heat management	0/1	1 and 2
Pig	Farm	Pasture use	Seasonal/all year round	2
Pig	Farm	Microregion	Boziu, Casinca, Alesani, Gravonna, Plaine Orientale, Cruzzini	1
Pig	Farm	Owner	6 owners anonymized	2
Pig or wild boar	Farm of hunting area	Area	6 areas for wild boars and 4 areas for pigs	2 and 3

## Results

### ADV and HEV Seroprevalence and Risk Factors in the Corsican Domestic Pig Population (Dataset 1)

A total of 213 domestic pigs were sampled at the slaughterhouse, but the analyses were performed on the 151 individuals for which there were no missing data for any of explanatory variables or serological assays. Average seroprevalence in this dataset was 41.7% (95% CI [33.8%; 49.6%]) for ADV and 85.4% (95% CI [79.8%; 91.0%]) for HEV. These values were higher in free ranging and fenced pasture farms than in closed farms [average prevalence was 58.4% (95% CI [48.8%; 68.0%]) and 93.0% (95% CI [88.1%; 97.9%]) in free ranging farms regarding ADV and HEV, respectively].

#### Major Risk Factors Associated with ADV Seropositivity in Pigs

We retained three potential best models according to their QAICc values (delta-QAICc < 2) (QAICc are detailed in the additional materials), but the significant average effects only concerned the vaccination treatment, sow castration and farm biosafety (full model-averaged coefficients detailed in Table 2a). The vaccination treatment was negatively correlated to ADV prevalence: a lower seroprevalence was observed in farms that implemented a self-vaccination process (OR_no vaccination/self-vaccination_ = 3.88, 95% CI [1.30; 11.57]), but this protective effect was much higher in farms that had implemented the official vaccination plan (OR_self-vaccination/official_ = 3.39, 95% CI [1.07; 10.75]). We also observed a protective effect of sow castration (OR_no castration/castration_ = 4.24, 95% CI [1.31; 13.71]). Traditional free ranging farms exhibited a higher seroprevalence compared with the closed ones (OR_free ranging/close_ = 6.14, 95% CI [1.11; 34.12]); the absence of ADV seropositive individual in fenced pasture farms in our sample could not allow the comparison of that particular category to other biosafety levels. All other effects were not significant and were finally not retained (Table 2a).

**Table 2:** Parameters estimates among the set of best models (model averaging) for dataset 1 (pigs sampled at the slaughterhouse at a large scale).

	Estimate	SE	Adjusted SE	*z*-Value	Pr (>|*z*|)
**(a) Regarding Aujeszky’s disease virus**					
**Vacc (no vs voluntary)**	**1.35680**	**0.55691**	**0.56158**	**2.416**	0.0157*
**Vacc (official vs voluntary)**	**−1.22555**	**0.58898**	**0.59395**	**2.063**	0.0391*
**Type (free ranging vs closed)**	**1.81566**	**0.87457**	**0.88168**	**2.059**	0.0395*
**Sows’ castration (yes)**	**−1.44539**	**0.59826**	**0.60331**	**2.396**	0.0166*
Type (open air vs closed)	−17.02230	1,279.67939	1,290.48297	0.013	0.9895
Age (young vs adult)	−0.15475	0.48168	0.48439	0.319	0.7494
Sex (male vs female)	−0.05546	0.23302	0.23456	0.236	0.8131

**(b) Regarding hepatitis E virus**					
**Type (fenced pasture and free ranging vs closed)**	**2.30771**	**0.68869**	**0.69288**	**3.331**	0.000867*
Sows’ castration (yes)	0.04097	0.23187	0.23342	0.175	0.860689
Age (young)	−0.36303	0.67060	0.67264	0.540	0.589398
Sex (male)	−0.06955	0.26421	0.26562	0.262	0.793440

*Significant p-value.

*Significant parameters for each data set are indicated in bold*.

#### Major Risk Factors Associated with HEV Seropositivity in Pigs

We retained four potential best models according to their QAICc values (delta-QAICc < 2) (additional materials), but the significant average effects only concerned the farm biosafety (full model-averaged coefficients detailed in Table 2b). Traditional free ranging farms and fenced pasture farms both exhibited a much higher seropositivity compared with closed farms, we finally merged both categories fenced pasture and free ranging categories since these two categories exhibited the same risk level (OR_free ranging or open air/closed_ = 10.05, 95% CI [2.61; 38.76]). Other effects were not significant and were finally not retained (Table 2b).

### Seroprevalence and Risk Factors in Domestic Pigs from Six Traditional Farms in Boziu-Verde (Dataset 2)

The analyses were performed on 75 pigs with no missing data for any of the explanatory variables and serological results. Average seroprevalence in this dataset was 48.0% (95% CI [36.7%; 59.3%]) for ADV and 30.7% (95% CI [20.3%; 41.1%]) for HEV.

#### Risk Factors Associated with ADV Seropositivity

We retained six potential best models according to their QAICc values (delta-QAICc < 2) (additional materials), but significant average effects only concerned pig reproductive status and management of sows in estrus (full model-averaged coefficients detailed in Table 3a). Reproductive pigs were more at risk than others (OR_breeder/fattening_ = 27.23, 95% CI [2.44; 303.68]) and the management of sows on estrus had a protective effect (OR_no management/mating management_ = 67.45, 95% CI [6.43; 707.43]). Other effects were not significant and were finally not retained (Table 3a).

**Table 3:** Parameters estimates among the set of best models (model averaging) for dataset 2 (free ranging pigs from Boziu Verde).

	Estimate	SE	Adjusted SE	*z*-Value	Pr (>|*z*|)
**(a) Regarding Aujeszky’s disease virus**					
**Sows mating management (yes)**	**−4.2114**	**1.1991**	**1.2180**	**3.458**	0.000545*
**Reproductive status (yes)**	**3.3042**	**1.2305**	**1.2473**	**2.649**	0.008071*
Sex (male)	1.8744	1.2264	1.2380	1.514	0.129999
Sero hepatitis E virus (HEV) (positive)	1.3607	1.4478	1.4569	0.934	0.350327
Zone 2 (vs Zone 1)	−0.0454	0.6389	0.6505	0.070	0.944364
Zone 3 (vs Zone 1)	5.9474	1,071.7341	1,091.4423	0.005	0.995652
Zone 4 (vs Zone 1)	0.2780	0.9535	0.9631	0.289	0.772872
Pasture use (seasonal vs permanent)	0.3833	1.0516	1.0627	0.361	0.718347

**(b) Regarding HEV**					
**Age (young vs adult)**	**−2.2175**	**0.7844**	**0.7956**	**2.787**	0.00532*
**Pasture use (seasonal vs permanent)**	**−2.0819**	**0.9163**	**0.9314**	**2.235**	0.02541*
Sows management (yes)	0.1078	0.3747	0.3784	0.285	0.77582
Sex (male vs female)	−0.3589	0.6538	0.6579	0.546	0.58538
Reproductive status (yes)	−1.5416	1.0227	1.0317	1.494	0.13511

*Significant p-value.

*Significant parameters for each data set are indicated in bold*.

#### Risk Factors Associated with HEV Seropositivity

We retained four potential best models according to their QAICc values (delta-QAICc < 2) (additional materials), but significant average effects only concerned pigs’ age and the intensity of natural pasture/forests use (full model-averaged coefficients detailed in Table 3b). Adult pigs were more at risk than young ones (OR_adult/young_ = 9.18, 95% CI [1.97; 42.73]), and farms permanently using natural pastures/forests showed a higher risk than farms only using them in autumn (OR_permanent/autumn_ = 8.02, 95% CI [1.33; 48.32]). Other effects were not significant and were finally not retained (Table 3b).

### Seroprevalence and Risk Factors of ADV and HEV in Wild Boar from Boziu-Verde (Dataset 3)

Out of 297 wild boars sampled, 274 analysis could be performed (23 samples could not be analyzed because of sample quality defaults). Average seroprevalence in this dataset was of 45.1% (95% CI [39.8%; 50.4%]) for ADV and 38.7% (95% CI [33.2%; 44.2%]) for HEV.

#### Risk Factors Associated with ADV Seropositivity

We considered three potential best models according to their QAICc values (delta-QAICc < 2) (additional materials), but the significant average effects only concerned wild boar’s age and wild boar seropositivity for HEV (full model-averaged coefficients detailed in Table 4a). Adult wild boars had a higher risk than young ones (OR_adult/young_ = 3.80, 95% CI [2.14; 6.77]) and HEV seropositive animals were more at risk than seronegative ones (OR_HEV+/HEV−_ = 2.06, 95% CI [1.51; 3.52]). Other effects were not significant and were finally not retained (Table 4a).

**Table 4:** Parameters estimates among the set of best models (model averaging) for dataset 3 (hunted wild boar from Boziu Verde).

	Estimate	SE	Adjusted SE	*z*-Value	Pr (>|*z*|)
**(a) Regarding Aujeszky’s disease virus (ADV)**					
**Age (young vs adult)**	**−1.144144**	**0.269294**	**0.270408**	**4.231**	2.32e−05*
**Sero hepatitis E virus (HEV) (positive)**	**0.701042**	**0.270076**	**0.271184**	**2.585**	0.00973*
Year (2014 vs other)	17.137850	912.961823	916.756557	0.019	0.98509
Sex (male vs female)	0.046974	0.148616	0.148972	0.315	0.75252
Hybrid status (yes)	−0.116537	0.248816	0.249249	0.468	0.64010

**(b) Regarding HEV**					
**Year (2014 vs other)**	**2.16122**	**0.78643**	**0.78969**	**2.737**	0.00620*
**Pig presence (permanent vs absent)**	**0.93853**	**0.32747**	**0.32876**	**2.855**	0.00431*
**Sero ADV (positive)**	**0.78865**	**0.28158**	**0.28269**	**2.790**	0.00527*
Pig presence (intermittent vs absent)	0.09843	0.35303	0.35450	0.278	0.78126
Age (young vs adult)	−0.47865	0.35488	0.35562	1.346	0.17832
Sex (male vs female)	−0.03197	0.12827	0.12863	0.249	0.80369
Hybrid status (yes)	−0.10445	0.24468	0.24512	0.426	0.67003

*Significant p-value.

*Significant parameters for each data set are indicated in bold*.

#### Risk Factors Associated with HEV Seropositivity

We considered 11 potential best models according to their QAICc values (delta-QAICc < 2) (additional materials), but the significant average effects only concerned the intensity of pig presence on natural pastures/forests, the year 2014, and wild boar seropositivity regarding ADV (full model-averaged coefficients detailed in Table 4b). The hunting areas with permanent pig presence showed the highest seroprevalence (OR_permanent/other_ = 3.63, 95% CI [1.12; 11.71]), while the seasonal use of pasture did not show higher seroprevalence than areas with no pig presence (*p* = 0.53). As previously observed, HEV and ADV serological status were correlated (OR_aujeszky+/aujeszky−_ = 2.36, 95% CI [1.37; 4.08]). Year 2014 appeared at risk compared with other years. However, this result relied on a very low sample on that year and might thus correspond to a particular family/spatial cluster rather than a real year effect (OR_2014/other_ = 9.07, 95% CI [1.92; 42.87]). Other effects were not significant and were finally not retained (Table 4b).

## Discussion

We explored seroprevalence regarding two infectious pathogens corresponding to different transmission patterns (ADV with direct transmission; HEV with direct and indirect transmission routes) at the interface of wild and domestic swine in Corsica. During the first step, we observed pigs’ seroprevalences at a large scale. Regarding both diseases, the high seroprevalence observed in all pig age classes and the absence of differences in seroprevalence among the different microregions confirmed the enzootic situation in Corsican pigs. Regarding ADV, our results confirmed the protective effect of vaccination. The official vaccination plan proposed by the French animal health authorities^1^ (35) between 2011 and 2013 is highly effective in comparison with farms that do no vaccinate their pigs and also compared with the partial protective effect of vaccination performed outside the official vaccination plan. Regarding both diseases, we observed a higher risk in free ranging pig farms (and open air fenced farms for HEV) than in intensive indoor ones, suggesting that the wild boar/pig interface and/or extensive farming practices in the contact with the natural environment might play a role in the exposure to both diseases.

Concerning free ranging pig farms, we confirmed the protective effect of limiting the release of sows during estrus on pastures for ADV seroprevalence (i.e., by mating sows before transferring them to natural pastures/forests), suggesting a risk linked to contacts with wild boars and highlighting the value of mating management limiting ADV transmission on free ranging farms. We also observed a higher ADV seroprevalence in breeder pigs than fattening ones, which may reflect the higher risk of breeders and their longer life compared with fattening pigs. These results are consistent with the expected sexual transmission pattern associated with ADV and the polygynous reproductive pattern observed in swine. The level of seroprevalence was similar in wild boars and pigs (i.e., 40–45%) and matched with previous studies performed in Corsica confirming the fact that ADV has been circulating for a long time in both wild and domestic populations (11, 12). This is one of the highest seroprevalence rates observed in wild boars in France (12) although wild boars are not as intensively managed in Corsica as in Central–Southern Spain (36, 37). We did not observe any additional risk in areas where free ranging pigs shared natural pastures/forest with wild boar, suggesting that the sylvatic and domestic epidemiological cycles are mostly independent even though the two populations are sympatric and sexual transmission is known to occur. In other European countries being in a situation of strict segregation, such as in Germany and continental Northern Italy, a complete asynchrony has been observed and different virus strains have been identified in the wild and domestic swine populations (38, 39). In our study, unmanaged domestic sows had a higher risk of becoming infected than other individuals, suggesting that sylvatic and domestic cycles are partly connected and that managing female in estrus limits the risk of ADV spill over. In addition, even if recent studies describe endemic patterns of ADV in wild boar populations, through female social behavior favoring intraspecific contacts (40, 41), further studies aiming at genotyping circulating virus strains, describing the space use and the genetic flow between domestic and wild populations are needed to better characterize these connections.

Regarding HEV, the study suggests a twofold lower exposure in wild boars (<40%) compared with domestic pig populations (>85%). The higher seroprevalence observed in domestic pigs suggests the spillover of HEV from pigs to wild boar rather than the contrary. However, since only wild boar populations from the Boziu-Verde were sampled in our study, we recommend a larger wild boar surveillance design, extended to other microregions combined with virus investigation and typing, to confirm this hypothesis. In free ranging farms (dataset 2), apparent seroprevalence (30%) was lower than expected when considering data set 1 (>90%); such a gap might correspond to a lower sensitivity of the serological ELISA test when using filter papers (used for dataset 1) instead of sera (used for dataset 2). The effect of domestic pig presence in hunting areas on wild boar seroprevalence suggests that inter-transmission does occur at the wild/domestic interface, possibly through environmental contamination and common attractors (e.g., contaminated water or food sources), as suggested by previous studies (16, 17). Nevertheless, the precise environmental risk factors (such as the role of water sources) for HEV transmission are still unclear and should be explored further in the future (42). Recent studies have suggested that hybrid wild boar populations might be more susceptible to certain infectious diseases than pure specimens (43, 44), and this association was also observed in Corsica when the prevalence of HEV was compared in populations of pure and hybrids wild boars (17). This potential role of hybrids was not confirmed by our analysis: once the use of natural habitats was taken into account, the hybrid status of wild boars was no longer significant, and we did not observe an additional risk in the domestic crossbred animals sampled in farms. These results suggest a confounding effect between animal population sympatry and the occurrence of hybrid individuals (these two factors being highly correlated) rather than a potential role of hybrids in HEV transmission between both populations. However, our data were based on phenotypical classification of hybrids which might not be precise enough to discern accurately between hybrid and pure individuals. Further studies using appropriate genetic methods to determine hybridization (45, 46) are needed to assess the hypothetical link between hybridization and disease susceptibility in Corsica.

Our results suggest that ADV and HEV infections can occur simultaneously in domestic pigs and wild boars. To the best of our knowledge, this potential co-infection has never been investigated in domestic or wild pigs. Recent studies have shown that a co-infection between HEV and other immunosuppressive porcine viruses such as PRSS can influence HEV infection dynamics concerning times of excretion or maintenance of the virus in the liver (47, 48). Since ADV can also affect the liver, a co-infection with both viruses could potentially increase liver damage, but further studies are needed to explore the pathogenic effects of co-infection with both viruses. Since no synergic mechanisms have been described between these two swine pathogens to date, we rather hypothesize a possible effect of local aggregations factors (e.g., family groups, water, or food sources) that might facilitate the transmission of both contagious diseases in wild boar [such as described by Acevedo et al. (36) and Vicente et al. (49) in other Mediterranean areas].

It is important to mention that, as our results are only exploratory since they are based on small samples and exposed to biases, they should be taken with caution; it is obvious that other non-considered factors unrelated to the wild/domestic interface might influence wild boar and domestic pig exposure to diseases. The effect of the year 2014, while seroprevalence in wild boar was stable all over the other years for both diseases, could be due to such uncontrolled factors or a sampling bias (50).

Finally, if the exploratory character of our study shows interesting results, it is essential to formulate research perspectives to confirm and improve our findings. We focused on one microregion only (Boziu-Verde), as a first step to test our hypothesis, and a comparative approach with other microregions, with different characteristics (microregions without pig farming activities, for example, or with a different distribution of technically advanced farms, or with less numerous wild board presence, etc.), is strongly needed. Another perspective is to combine this type of study with molecular approaches to identify pathogen strains in wild boar and pig populations. In the case of HEV, several recent studies have shown that several strains are shared between pigs, wild boars, pork products, and humans (16, 51). Such approaches can be relevant to address the question of environmental contamination (52). Concerning ADV, a major issue is the technical difficulty to collect samples containing the virus, as Aujeszky’s disease symptoms are difficult to notice in free ranging farming systems.

## Conclusion

To the best of our knowledge, this study represents the very first attempt to assess the use of disease seroprevalence values as indicators of the importance of wild boar/domestic pig interactions and the potential efficacy of disease management strategies for preventing disease maintenance and spread in an extensive pig farming environment such as the one occurring in Corsica. It provides evidence of the strong protective effect of ADV vaccination, which should encourage farmers to commit to future vaccination programs. Moreover, we provide evidence of the benefits of reproductive management of sows (i.e., spaying or mating before release them into natural habitats). In the future, the cost-effectiveness of such measures on farm productivity (e.g., average litter size or pig growth rate) needs to be quantified. Our study highlighted a limited protective effect of pig farm biosecurity measures toward both diseases, suggesting that the wild/domestic interactions are possibly not the only one and nor the most important factor explaining disease dynamics in Corsican pigs. Our study provides evidence of the connection between wild and domestic disease cycles on traditional free ranging farms and the usefulness of promoting seasonal partial segregation of the swine populations. However, awareness needs to be raised among farmers and hunters on the likely presence of other unknown/uncontrolled factors unrelated to the wild/domestic pig interface. Therefore, further analysis and confirmation of these identified trends is recommended to explore the potential impact of other factors affecting the transmission of those pathogens and to better understand their dynamics and the impact of management measures.

## Ethics Statement

The study was exempt of authorization because the serological analysis were carried out on blood samples collected either on live pigs by veterinarian technicians at the farm level or at the slaughterhouse as part of a surveillance scheme, or on shot wild boars by hunters.

## Author Contributions

The two first authors contributed equally: FCharrier and SR. Conceived and designed the seroprevalence studies: FCharrier, OM, and FCasabianca. Performed the studies: FCharrier, FJ, OM, and FCasabianca. Analyzed the data: FCharrier, SR, FJ, CR, and FCasabianca. Carried out the statistical analyses: SR. Contributed reagents/materials/analysis tools: FCharrier, OM, FCasabianca, NP, M-FL, and JJ. Wrote the paper: SR and FCharrier. Critically read the manuscript: FJ, CR, CD, NP, and M-FL. Read and approved the final manuscript: FCharrier, SR, FJ, OM, CR, FCasabianca, CD, NP, and M-FL.

## Conflict of Interest Statement

The authors declare that the research was conducted in the absence of any commercial or financial relationships that could be construed as a potential conflict of interest.

## Footnotes

^1^Fédération Régionale des Groupements de Défense Sanitaire de Corse (FRGDSB20), 2014. Plan Expérimental de lutte contre la maladie d’Aujeszky—Bilan après trois années de mise en œuvre. Technical report presented in Ajaccio, on the 19th of September 2014. This report presents the technical results of the Aujeszky disease management strategy implemented by Health Authorities in Corsica from 2011 to 2014.
